# Direct RNA sequencing enables m^6^A detection in endogenous transcript isoforms at base-specific resolution

**DOI:** 10.1261/rna.072785.119

**Published:** 2020-01

**Authors:** Daniel A. Lorenz, Shashank Sathe, Jaclyn M. Einstein, Gene W. Yeo

**Affiliations:** 1 Department of Cellular and Molecular Medicine, University of California San Diego, La Jolla, California 92093, USA; 2 Stem Cell Program, University of California San Diego, La Jolla, California 92093, USA; 3 Institute for Genomic Medicine, University of California San Diego, La Jolla, California 92093, USA

**Keywords:** m^6^A, nanopore, RNA modifications

## Abstract

Direct RNA sequencing holds great promise for the de novo identification of RNA modifications at single-coordinate resolution; however, interpretation of raw sequencing output to discover modified bases remains a challenge. Using Oxford Nanopore's direct RNA sequencing technology, we developed a random forest classifier trained using experimentally detected *N*^6^-methyladenosine (m^6^A) sites within DRACH motifs. Our software MINES (m^6^A Identification using Nanopore Sequencing) assigned m^6^A methylation status to more than 13,000 previously unannotated DRACH sites in endogenous HEK293T transcripts and identified more than 40,000 sites with isoform-level resolution in a human mammary epithelial cell line. These sites displayed sensitivity to the m^6^A writer, METTL3, and eraser, ALKBH5, respectively. MINES (https://github.com/YeoLab/MINES.git) enables m^6^A annotation at single coordinate–level resolution from direct RNA nanopore sequencing.

## INTRODUCTION

Since the identification of the first RNA modification more than 60 years ago, more than 100 different RNA modifications have been identified (Davis and Allen 1957; Jonkhout et al. 2017). These RNA modifications are capable of imparting new or altered functions in RNA and have since been collectively termed the epitranscriptome (Saletore et al. 2012). One of the most common modifications in the eukaryotic transcriptome is *N*^6^-methyladenosine (m^6^A), which is found in most classes of RNA, including mRNA, ncRNA, rRNA, and tRNAs (Deng et al. 2018; Ma et al. 2018). With the development of antibodies that recognize m^6^A and coupling to high-throughput sequencing technologies, several transcriptome-wide approaches for identifying m^6^A sites have been developed (Grozhik and Jaffrey 2018). These techniques have been useful in demonstrating that m^6^A plays important roles in nearly every aspect of biology from yeast to mammals (Yue et al. 2015).

Biochemical studies have revealed a complex network of proteins that are involved in writing, reading, and erasing m^6^A methylation. In humans, current evidence suggests that a complex, composed of proteins METTL3, METTL14, and WTAP, is responsible for installing the m^6^A modification in most mRNAs (Liu et al. 2014). These sites are then recognized by several families of proteins including YTH-domain-containing, IGF2BP (IMPs), and HNRNP proteins, each having uniquely characterized roles in reading m^6^A, influencing processes such as splicing, transcript stability, and localization (Shi et al. 2019). m^6^A modification is a dynamic process and can be removed or “erased” by demethylases, ALKBH5 and FTO. Dysregulation of any of these critical proteins results in changes to m^6^A levels and has been linked to a myriad of diseases, including cancer and neurological diseases (Chen et al. 2019; Delaunay and Frye 2019).

Although second-generation polymerase-based sequencing has enabled transcriptome-wide studies of RNA biology, new third-generation sequencing is being developed to overcome limitations such as amplification biases, lack of single-molecule sensitivity, and isoform ambiguity. One of these methods, commercialized by Oxford Nanopore Technologies (ONT), uses nanopore-based sequencing to detect changes in electric current as a single strand of nucleic acid sequence transverses a pore protein. By deconvoluting these electrical signals, the specific nucleotide sequence can be reconstructed. This technology offers long sequencing reads (up to 2 Mb) and detection of epigenetic markers (Payne et al. 2019). To illustrate, nanopore-based DNA sequencing has been able to detect the endogenous DNA modifications, m^5^C and m^6^A (Simpson et al. 2017; McIntyre et al. 2019). Recently, ONT reported the first RNA-sequencing method capable of directly sequencing individual RNA strands while preserving epitranscriptomic information using fully modified in vitro transcribed RNAs; however, single-molecule detection remains problematic because of the one in 13 single-base error rate (Garalde et al. 2018).

Here we evaluate the ability of nanopore-based sequencing to directly detect m^6^A RNA modifications in endogenous transcripts, providing numerous benefits over traditional methodologies including single-coordinate-level resolution, isoform-specific context, single experimental pipeline, and simplified bioinformatic detection. Based on changes observed in the current signal from each site, MINES is able to predict known m^6^A CLIP-seq sites with ∼80% accuracy within certain DRACH sequences that represent ∼35% of reported CLIP sites. When applied to RNA from a primary human mammary epithelial cell line (HMEC), MINES identified 42,116 m^6^A sites at single-coordinate and isoform-level resolution. As nanopore-based sequencing becomes ubiquitous in RNA-seq studies, our approach will facilitate new discoveries regarding m^6^A biology and serves as a useful framework for analyzing other RNA modifications using direct RNA sequencing.

## RESULTS

### DRACH filtering is required for de novo detection

**FIGURE 1.**
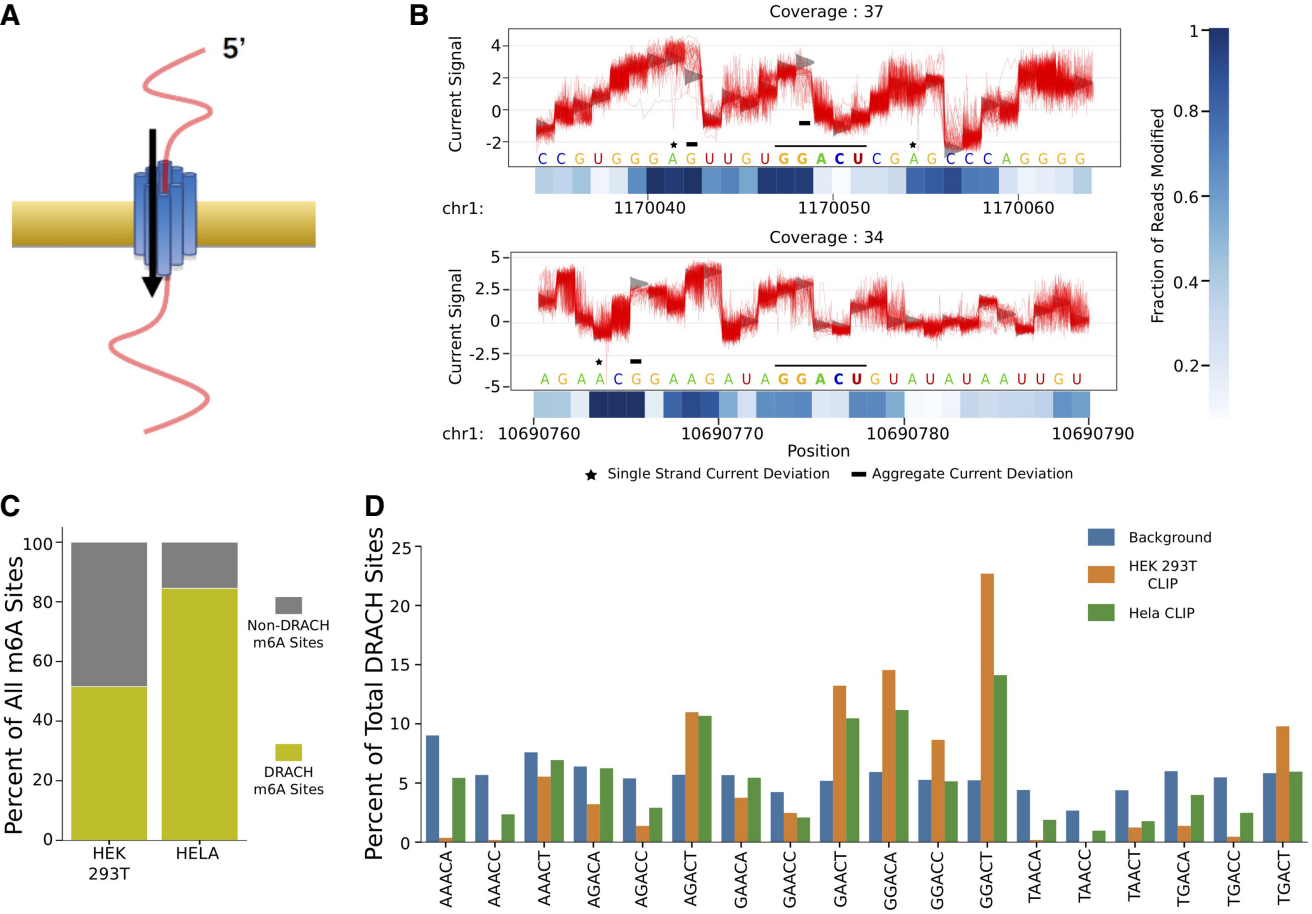
Filtering by DRACH motifs encompasses the majority of m^6^A sites. (*A*) Schematic of Nanopore-based sequencing. (*B*) Representative Tombo outputs depicting individual reads as red lines and expected values as gray distributions. The black bar in the *middle* highlights the GGACT motif. The heatmaps *under* each plot show Tombo's fraction modification value for each base. (*C*) Motif analysis of sites in HEK293T and HeLa cells from m^6^A CLIP data sets. (*D*) Bars representing the percentage of each DRACH motif in m^6^A CLIP and its relative enrichment over non-CLIP sites.

Nanopore-based sequencing is distinct from polymerase-based sequencing in that it can preserve and detect nucleic acid modifications as a single strand of nucleic acids passes through a pore (Fig. 1A). With the advent of commercially available direct RNA sequencing, we sought to detect one of the most abundant RNA modifications, m^6^A, on cellular transcripts. A recent study suggests direct sequencing can distinguish fully modified m^6^A sites in pure populations of synthetic RNAs from unmodified positions (Garalde et al. 2018). However, these recent methods are limited by the computational resources necessary to detect changes in raw current on a transcriptome-wide scale and have not yet been utilized to identify new endogenous m^6^A sites (Garalde et al. 2018; Workman et al. 2018; Liu et al. 2019). Contemporaneously, software applications, such as ONT's Tombo, enable detection of RNA modifications by determining a modification value from calculating the difference between the observed current and a ground truth provided by the reference genome. The fraction modification value is stored as site averages instead of a per read value to reduce the computational load. However, a challenge associated with all nanopore-based approaches centers around a 1:13 error rate (Depledge et al. 2019). Hence, relying solely on the “error detection” of de novo predictions from Tombo is unreliable at this time and prevents accurate single-molecule detection. This is highlighted in Figure 1B, with many sites exhibiting aggregate (black bars) and molecule-specific (black stars) deviations from the expected current values. To overcome this limitation while simultaneously maintaining a low computational burden, we reasoned that filtering nanopore data based on the known m^6^A DRACH motifs would be a pragmatic strategy for m^6^A detection. By limiting our algorithm to DRACH sites, we improve the likelihood that our predictions are specific to m^6^A sites and not to other mRNA modifications. Analysis of two site-specific m^6^A cross-linking and immunoprecipitation sequencing (CLIP-seq) data sets from HEK293T and HeLa cells (Linder et al. 2015; Ke et al. 2017) revealed that >50% and >80% of sites were located within DRACH sequences, respectively (Fig. 1C). Deeper analysis revealed that the most common pentamers present within the DRACH motif in both data sets is GGACT, with six sequences (AGACT, GAACT, GGACA, GGACC, GGACT, TGACT) representing >50% of CLIP sites within DRACH sequences (Fig. 1D). Thus, our strategy of prefiltering nanopore reads to reduce the computational load still encompasses the vast majority of m^6^A sites.

### Nanopore sequencing distinguishes m^6^A within DRACH motifs

**FIGURE 2.**
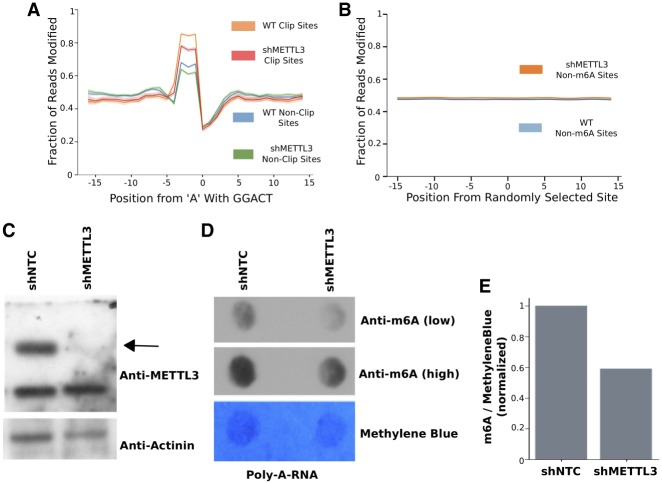
Nanopore sequencing can detect endogenous m^6^A. (*A*) Line plots depicting the mean Tombo's fraction modified value across a 30-nt window centered on the “A” in GGACT across all sites in RNA form HEK293T or shRNA targeting METTL3 (shMETTL3) cells. (*B*) Line plots of Tombo's fraction modified values across shuffled non-DRACH sites. (*C*) Western blot showing knockdown of METTL3 relative to nontargeting controls. The black arrow indicates expected METTL3 molecular weight. (*D*) m^6^A dot blot of poly(A) RNA from HEK293T cells treated with shNTC or shMETTL3. Methylene blue was contrast adjusted to highlight dots. (*E*) ImageJ quantification and normalization of *D*.

To evaluate the utility of our strategy, we sequenced poly(A)-selected RNA from HEK293T cells. Reads were aligned to the human hg19 reference genome. It should be noted that using a genomic reference in Tombo will currently only yield coverage along the 3′ untranslated regions (UTRs) as Tombo aligner is not splice-aware. Hence, our initial analyses were limited to alignments within the 3′ UTR but still comprise >40% of known m^6^A sites (Linder et al. 2015; Yue et al. 2015). This limitation can be surpassed by using a cDNA reference. From Tombo's de novo detection algorithm we collected the fraction modification values for all genomic positions within 3′ UTRs. The current pore protein used by Oxford Nanopore detects an ∼5-bp window. We therefore extended our input window to 30 bp centered on the “A” in the DRACH motifs to ensure detection of the site and flanking regions. Each window was labeled with a ground truth based on whether the midpoint site was found overlapping any site within the m^6^A CLIP-seq data sets. We required that each window must have a minimum read coverage of five reads, because of the error rate at low coverage loci. Even with this filtering, output fraction–modified values averaged ∼0.5 across all windows. The aggregate modification value was obtained for each coordinate within each window, and a spike in signal value was observed at positions 1 through 3 upstream of the GGACT motif compared to a randomly selected background (Fig. 2A,B). A similar spike was observed for AGACT, GGACA, and GGACC motifs as seen in Supplemental Figure 1, along with other DRACH motifs. Encouraged by a significant difference between sites with CLIP evidence relative to non-CLIP sites, we sought to confirm that the spike in signal was indeed due to m^6^A. To accomplish this, we generated a HEK293T cell line stably expressing a shRNA that successfully depletes METTL3 protein (Fig. 2C–E) and sequenced poly(A) RNA with ONT. METTL3 depletion had a greater effect on m^6^A levels in total RNA relative to the poly(A) fraction (Supplemental Fig. 2). A decrease in peak intensity was observed in the METTL3 shRNA cell line along the corresponding positions of the modified sites identified in the WT cell line. However, a similar change was not observed for randomly selected non-DRACH sites, indicating that the peak is indeed a result of the m^6^A methylation status (Fig. 2A,B). The METTL3 shRNA cell line also served as a validation for the sites identified in the WT cell line, independent of CLIP-based methods. Intriguingly, we found a similar decrease in peak intensity in both CLIP and non-CLIP sites, suggesting that there was a significant number of additional m^6^A sites that were likely undetected within the previous CLIP data sets (Fig. 2A).

### Random forest model predicts m^6^A sites

**FIGURE 3.**
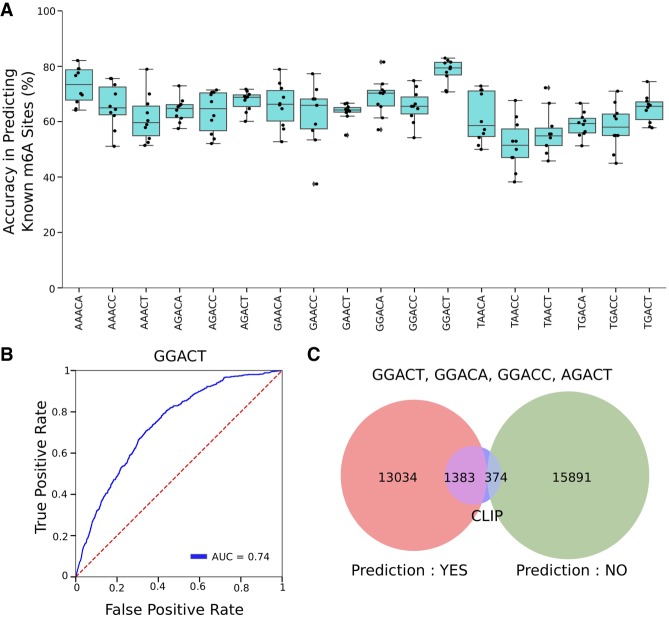
A trained RFM accurately predicts m^6^A within DRACH motifs. (*A*) Box plots showing the model's accuracy of predicting CLIP sites, organized by each DRACH motif across 10 training runs. (*B*) ROC curve for GGACT motif from the final model. (*C*) Venn diagram for the prediction of AGACT, GGACA, GGACC, and GGACT sites. CLIP sites represent data withheld from training for testing purposes.

After confirming that ONT is able to detect m^6^A sites that were novel as well as ones previously found by CLIP-based methods, we elected to use a random forest model (RFM) to predict methylation sites de novo (Pedregosa et al. 2011). The RFM was trained using 70% of the CLIP sites (positive labels) and an equal number of non-CLIP sites (*n* = 1450 for GGACT) as negative examples. The remaining 30% of CLIP sites were reserved as test examples. The test data also contained the remaining non-CLIP sites that were not included in the training data set. Because nanopore sequencing shows a unique sensitivity for each 5mer, we generated a separate model for each 5mer within the DRACH motif. We generated 10 models per DRACH motif based on random samples of training data and stored the model with the highest accuracy. Final accuracy values, defined as correctly predicted CLIP sites in the test data, ranged from 67% to 83%, whereas the precision values ranged from 40% to 92% (Fig. 3A; Supplemental Table 1). Area under the curve (AUC) values ranged from 0.54 to 0.76; however, we believe these values were negatively affected by the presence of novel, non-CLIP m^6^A sites (true negatives) within the test data set (Figs. 2A, 3B; Supplemental Fig. 3). Of the 18 DRACH motifs, only four generated models with accuracy >0.7, precision values >0.85, and ROC AUC values >0.67. Combining the four top motifs, the average accuracy was 79%, which represents >35% of known (CLIP-based) m^6^A sites (Fig. 3C). Interestingly, RFMs from motifs not meeting our accuracy, precision, and ROC AUC standards also clearly failed to exhibit a decrease in signal in the METTL3 knockdown data set at m^6^A CLIP sites (Supplemental Fig. 1). This either indicates that the current pore protein is incapable of distinguishing m^6^A methylation in these motif contexts or that these sites could represent off-target antibody binding or exists in such low m^6^A /A ratios that we are unable to detect their change in signal.

### Detection of novel m^6^A sites in HEK293

**FIGURE 4.**
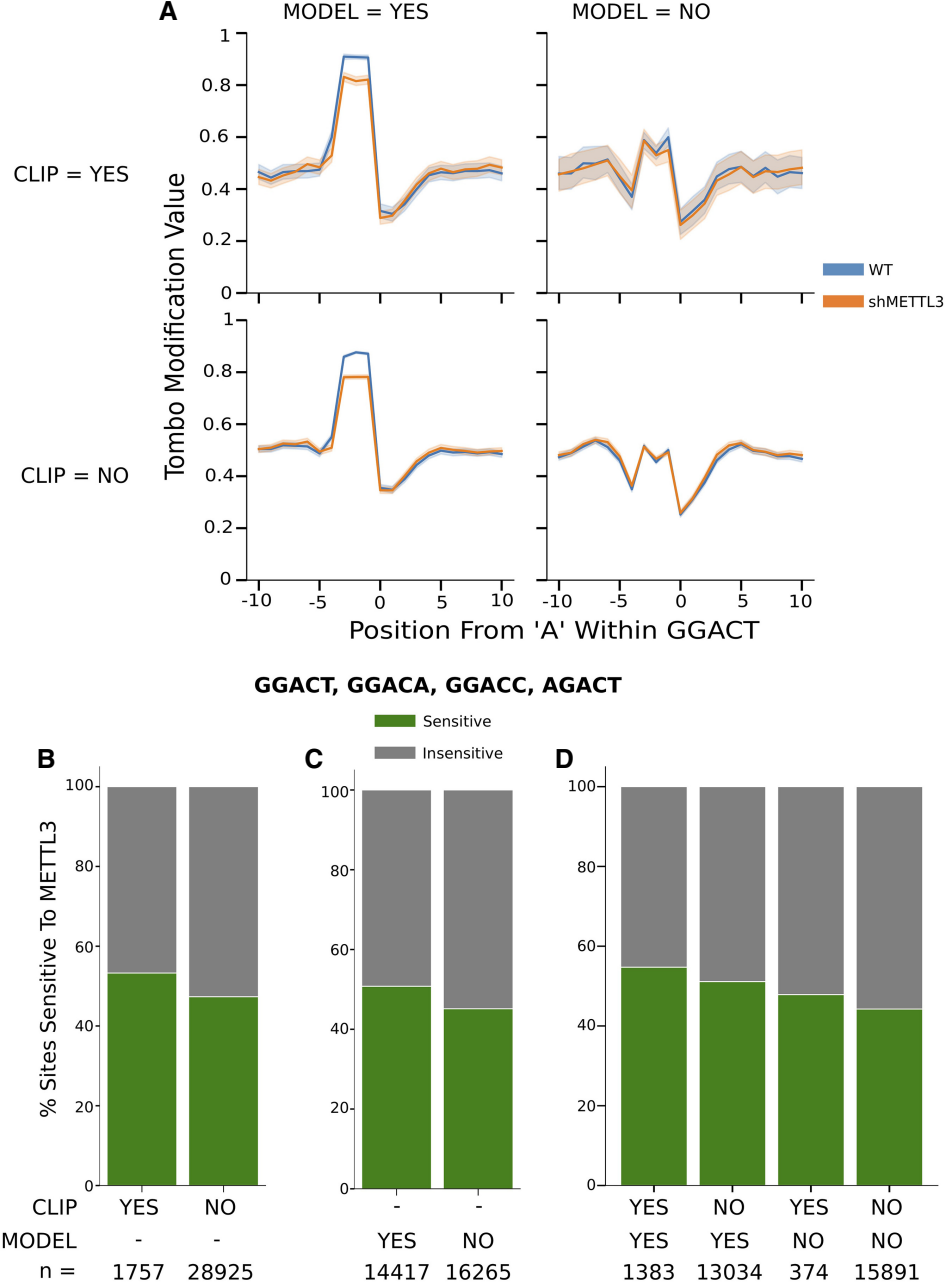
MINES-predicted sites mimic m^6^A CLIP sites. (*A*) Line plots of Tombo's fraction modified values broken down by CLIP sites and model predictions for GGACT in untreated HEK293T cells or HEK293T cells treated with shRNA targeting METTL3 (shMETTL3). (*B*–*D*) Percent of predicted m^6^A sites sensitive to METTL3 knockdown within the AGACT, GGACA, GGACC, and GGACT motifs.

Having generated a nanopore-enabled m^6^A detection algorithm, MINES, we evaluated the non-CLIP sites and predicted their methylation status. Of the 28,925 non-CLIP sites across AGACT, GGACA, GGACC, and GGACT motifs, MINES predicted that 13,034 are likely methylated (Fig. 3C). Surprised by the number of potentially missed m^6^A sites, we analyzed the mean modification values for these predicted sites in both wild-type and METTL3 knockdown (Fig. 4A; Supplemental Fig. 4). As expected, these sites displayed a peak in modification values that significantly decreased under METTL3 knockdown. This is in concordance with the CLIP sites correctly identified within the test data (true positives). This effect was not observed in the sites predicted to be unmodified, irrespective of whether they were previously identified from the m^6^A CLIP experiments (Fig. 4A, right panels). All other 5mers can be found in Supplemental Figure 4. To further characterize the wild-type peak sites, we looked at their response to METTL3 depletion on a per site basis. A METTL3-sensitive site was defined as any site with a greater mean modification value at the wild-type peak positions over METTL3 depletion. Figure 4B and C show the fraction of predicted m^6^A and non-m^6^A sites sensitive to METTL3 depletion mimics that of the CLIP data with a breakdown of each category in Figure 4D. Thus, this provides more evidence that MINES is correctly predicting m^6^A sites, as the number of sites sensitive to METTL3 increases to a similar degree as the CLIP sites.

### Cell line–independent detection and validation by ALKBH5 expression

**FIGURE 5.**
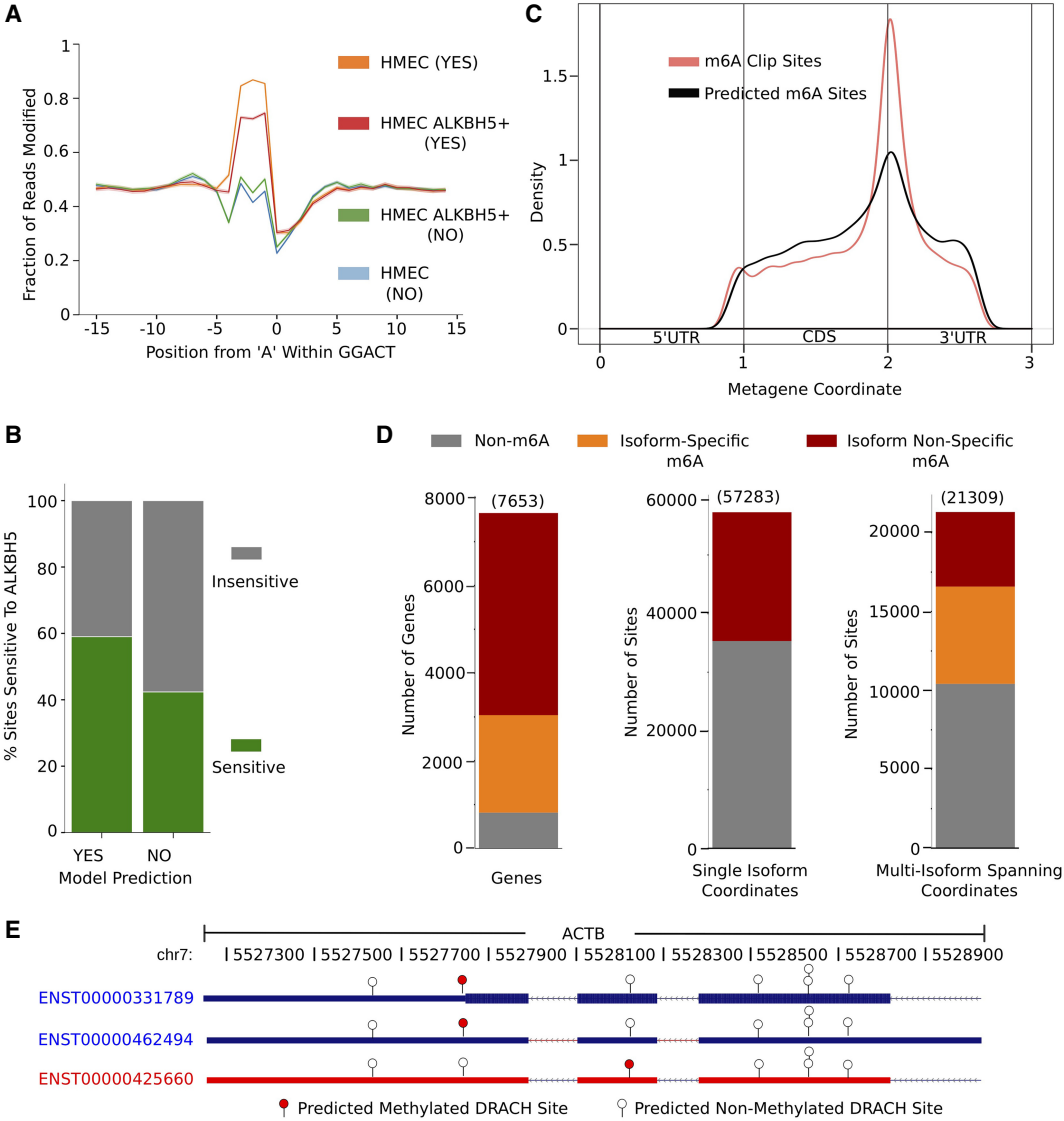
MINES is cell line–independent and provides isoform-level resolution. (*A*) Line plot of Tombo's fraction modified values in HMEC for GGACT and their m^6^A prediction status. (*B*) Percent of predicted m^6^A sites sensitive to ALKBH5 overexpression within the AGACT, GGACC, GGACT, and GGACA motifs. *n* = 42,116 (yes) and 71,365 (no). (*C*) Metagene analysis of m^6^A sites in HMEC within the AGACT, GGACC, GGACT, and GGACA motifs. (*D*) Bar plots summarizing MINES’ predictions with gene- and isoform-level resolution. (*E*) MINES isoform-level prediction of ACTB. Converted to hg38 coordinates.

To test whether our model is able to detect m^6^A-modified sites in other cell lines, we sequenced poly(A) RNA from a primary HMEC and a derivative cell line that stably overexpresses the m^6^A eraser ALKBH5. Decreased m^6^A levels due to ALKBH5 overexpression were confirmed by western and dot blot analyses (Supplemental Fig. 5A,B). Here, we aligned sequencing reads to a human cDNA reference to ensure full transcript coverage and evaluated the ability of MINES to predict m^6^A in isoform-specific levels. Using Tombo's coverage data and fraction modified values, and the RFMs generated for four motifs (AGACT, GGACA, GGACC, GGACT), MINES assigned m^6^A status to 42,116 sites. Similar to the HEK293T and METTL3 knockdown results, the mean modification values for the HMEC m^6^A sites (true positives) were lower in the ALKBH5 overexpression cell line (Fig. 5A; Supplemental Fig. 6) compared to randomly shuffled sites (Supplemental Fig. 5C). Some DRACH sequences produced altered modification patterns than those found in Supplemental Figure 1; however, these are limited to sequences in which the accuracy and precision were poor and are not included in the final versions of MINES. The fraction of individual sites sensitive to ALKBH5 also increased in the m^6^A predicted fraction (Fig. 5B), similar to METTL3 knockdowns. To further assess the accuracy of MINES, we studied the distribution of predicted m^6^A sites across all transcript isoforms to resolve the density of m^6^A sites within different genic regions including 5′ UTR, CDS, and 3′ UTR, respectively (Fig. 5C). This analysis revealed the characteristic density peak at the start of the 3′ UTR, confirming that our model resembles results seen in traditional m^6^A-seq approaches(Linder et al. 2015; Ke et al. 2017).

To determine differential isoform-level methylation patterns, we converted the cDNA coordinates to genomic positions. Analysis of these genomic positions identified 2225 genes to have isoform-specific methylation patterns out of the 6837 m^6^A-containing genes (Fig. 5D). In total there were 78,592 distinct genomic locations analyzed by MINES with 21,309 of these positions covering multiple isoforms. Comparing the methylation status of these multiple isoform sites revealed 10,415 sites that were never predicted to be methylated, 4726 sites predicted to be consistently methylated, and 6168 sites with isoform-specific methylation (Fig. 5D). As an example, we looked at three ACTB isoforms that were found in our nanopore sequencing data and predicted by MINES to have isoform-specific m^6^A. The three isoforms (ENST00000331789, ENST00000425660, and ENST00000462494) had seven sites that met our read depth and sequence requirements (Fig. 5E). Two of the transcripts (ENST00000331789 and ENST00000462494) were predicted to contain one m^6^A site at genomic position chr7:5527743 (hg38). The third transcript (ENST00000425660) is not methylated at this position but was instead predicted to be methylated at chr7:5528125 (hg38). Intriguingly, this third transcript is also predicted by ENSEMBL annotation to be subject to nonsense-mediated decay; however, future experiments would be required to link these events. It should be noted that this isoform-level resolution is only possible if a cDNA reference was used as input to Tombo to perform the read alignment. Thus, MINES, for the first time, enables probing of m^6^A biology with isoform-specific resolution.

## DISCUSSION

Although effective, m^6^A CLIP-seq and RIP-seq techniques depend on the availability of high-quality antibodies and require longer library preparation times and tailored processing pipelines for analysis. Advances in third-generation sequencing approaches have enabled direct RNA sequencing while preserving endogenous modifications with a short and straightforward library preparation and isoform-specific detection. Taking advantage of this recent technology we developed an algorithm that uses only the standard data generated from an ONT sequencer as input and predicts m^6^A modified sites in poly(A) selected mRNA.

Coupling publicly available m^6^A data sets and Tombo's modification values, we demonstrated a largely accurate detection of m^6^A sites at positions 1 through 3 upstream of canonical DRACH motifs. Through the sequencing of a METTL3 knockdown cell line, we showed that the modification value decreases at these previously reported sites, whereas randomly selected background sites remain unaffected. This serves as an independent validation of our results. Interestingly, we observed a decrease in modification value in several non-CLIP sites upon METTL3 knockdown, indicating potentially unannotated m^6^A sites. We then trained an RFM using the CLIP sites as positive controls and non-CLIP sites as negative controls. Four DRACH sequences (AGACT, GGACT, GGACC, and GGACA) generated models with maximum accuracy >70% and precision >85%, comprising >35% of known m^6^A sites. Using MINES to identify methylation sites within these sequences, we predicted a total of 13,034 m^6^A sites in HEK293T cells. These newly identified sites exhibited similar modification values and sensitivity to loss of METTL3 to those found in previous data sets. Factoring in the low individual base accuracy and high computational burden of analyzing signal deviations for each RNA molecule, we elected to use average deviations for each site and therefore cannot accurately determine the percentage of reads methylated at a given site at this time. Additionally, this averaging could result in the loss of methylated sites with low m^6^A/A ratios, as small differences could be lost to background. As improvements to the pore protein are released in the future, MINES can be easily retrained to achieve single-molecule-level detection.

Next, we utilized MINES to identify and annotate 42,116 m^6^A sites in an HMEC line. As supporting validation of these sites, we generated a cell line that overexpresses ALKBH5. These newly annotated sites showed a significant increase in ALKBH5 sensitivity over nonmethylated sites, consistent with our results in the METTL3 depletion in HEK293. These new sites also mimic the distribution of m^6^A sites in other cell types with a characteristic peak at the beginning of the 3′ UTR, immediately following the stop codon. Using cDNA alignments, MINES was able to predict m^6^A methylation in an isoform-specific manner for 2225 genes (Fig. 5D), illustrated in Figure 5E with ACTB. Thus, we are confident in MINES’ ability to annotate m^6^A sites in any transcriptome with isoform-level resolution using raw nanopore data as input. We envision this method and software to be readily adopted in the current m^6^A detection field.

## MATERIALS AND METHODS

### Cell line generation and culture

HMECs expressing hTERT and tamoxifen inducible Myc-ER (Myc-ER-HMECs) were a gift from Trey Westbrook (Kessler et al. 2012). HEK293 and HMEC cell lines were cultured in DMEM supplemented with 10% FBS and Medium 171 supplemented with MEGS: S0155, respectively, following standard tissue culture practices. METTL3 shRNA plasmid (TRCN0000034717) was purchased from Sigma-Aldrich. psPAX.2 and pMD2.g were a gift from Didier Trono (Addgene plasmids #12260, #12259). ALKBH5 was cloned from endogenous HMEC cDNA into doxycycline-inducible pLIX403 with a carboxy-terminal mRuby tag using Gateway assembly. pLIX403 was a gift from David Root (Addgene plasmid #41395). All plasmids were confirmed with Sanger sequencing. Briefly, lentivirus was packaged in HEK293T cells by seeding six-well plates at ∼80% confluence. The following day the cells were transfected by combining 35 µL Opti-MEM, 5 µL P3000 reagent (both Thermo Fisher), 500 ng psPAX.2, 50 ng pMD2.g, and 500 ng shRNA/gene vector. Then, 15 µL Opti-MEM and 4 µL Lipofectamine 3000 (both Thermo Fisher) were mixed in another tube before being combined together and allowed to incubate at room temperature for 20 min. This mixture was added to cells in a dropwise fashion. After 4–6 h, the media was replaced with fresh media. Media containing virus was harvested 48 and 72 h posttransfection. Viral particles were passed through a 0.45-µm sterile filter. Virus containing media was then added to HEK293T or HMEC cell lines supplemented with 8 µg/mL polybrene. Media was removed after 24 h and replaced with media containing 2 µg/ml puromycin. ALKBH5 overexpression was induced with the addition of 1 µg/mL doxycycline to media for 48 h before collecting cells.

### Western blots

Cell lysates were harvested at ∼80% confluency by washing with phosphate-buffered saline (PBS) and ∼150 µL lysis buffer (50 mM Tris-HCl pH 7.4, 100 mM NaCl, 1% NP-40, 0.1% SDS, 0.5% sodium deoxycholate) was added. Samples were sonicated, loaded on 4%–12% Bis-Tris gel, and transferred to PVDF membrane overnight at 30 V at 4°C. The membrane was then blocked with 5% nonfat dry milk powder in Tris-buffered saline with 0.05% Tween-20 (TBST) for 1 h, incubated with antibody (METTL3—Proteintech #15073-1-AP, ALKBH5—MBL #RN122PW, Actinin—Millipore #05-384, GAPDH—Abcam #ab8245) at 1:1000 dilution for 1 h, washed 3× with TBST, and incubated for 1 h with HRP-conjugated anti-rabbit (Thermo Fisher #31460) or anti-mouse antibody (Thermo Fisher #31430) at 1:3000 dilution before being washed again 3× with TBST. Bands were visualized by enhanced chemiluminescence (Thermo Fisher #34096) and exposure to film.

### RNA isolation and poly(A) selection

At 80% confluency in 10-cm plates, cells were washed with PBS and harvested in 1 mL of TRIzol reagent (Thermo Fisher) or Direct-zol kit with DNase treatment (Zymo Research). Total RNA was extracted following the manufacturer's protocol. Then, 20 µg of total RNA was poly(A)-selected using a poly(A) magnetic resin kit (NEB E7490L). RNA was then analyzed by high-sensitivity RNA TapeStation (Agilent #5067-5579) to confirm poly(A) selection and RNA quality.

### m^6^A dot blot

RNA was quantified prior to blotting using a Nanodrop spectrophotometer. Unless otherwise noted, 500 ng of RNA was then diluted to 100 µL in H_2_O and spotted on a prewashed (100 µL H_2_O) nylon membrane (Hybond-XL, GE Healthcare) using a dot blot apparatus (Bio-Dot, Bio-Rad) and washed with 100 µL of H_2_O. RNA was then cross-linked to the membrane with a UV cross-linker fitted with 254 nm bulbs at 120 mJ/cm^2^. The membrane was processed and developed as described above, using an m^6^A antibody (Synaptic Systems #202111) at 1:1000 dilution. After developing, the membrane was washed 3× with TBST, and methylene blue solution (0.04% methylene blue in 50 mM NaOAC, pH 5.0, Santa Cruz Biotechnology sc-215381) was added and allowed to rotate overnight. The following day the solution was removed, and the membrane was rinsed with 50% ethanol/water before being imaged. Dots were quantified by densitometry using ImageJ.

### Nanopore sequencing

Five hundred nanograms of poly(A)-selected RNA was used as input for the Nanopore direct RNA sequencing kit (SQK-RNA001 and 002). RNA was prepared following the manufacturer's protocol. Sequencing was carried out on an Oxford Nanopore Minion-101B using R9.4.1 flow cells for ∼48 h. Data was base-called in real time using a Dell Precision 7820 Tower with either Albacore or Guppy base callers. Total reads (in millions) were HEK-WT = 1.45, HEK-shMETTL3 = 1.1, HMEC-WT = 2.14, HMEC-ALKBH5 overexpression = 1.72.

### Tombo alignment and values

Reads and modification values were aligned using the default *resquiggle* and de novo detection settings, respectively, in Tombo v1.4 with hg19 and GRCh38/hg38 references using either a genomic or a cDNA (transcriptomic) reference. Genomic reference (hg19) was downloaded from GENCODE, and cDNA reference (GRCh38/hg38) was downloaded from Ensembl. WT HEK293T RNA was aligned to a custom hg19 reference containing an additional unique gene; reads mapping to this custom gene were not used. Values were obtained from the read coverage (bedgraphs) and the fraction of modified reads (wiggle files) for each position within the reference.

### m^6^A site detection using random forest models

Briefly, all regions within the reference containing a DRACH motif were identified and a new set of regions was generated by extending 10 bp on both sides of the “A” within the DRACH motifs. These regions were further filtered to have a minimum coverage of five reads. The DRACH regions were intersected with known m^6^A sites to identify true positive regions obtained from GSA data sets GSM1556678 and GSM2300429 REFs: PMID: 26121403, PMID: 28637692).

A random forest classifier is a decision tree–based classifier. The Python implementation of random forest (*sklearn)* was used to generate a model to predict m^6^A sites from the filtered DRACH data. Since Nanopore data reflects the occurrence of a m^6^A site with a change in aggregate modification values, we trained the random forest model on the change in corresponding modification values detected by Nanopore sequencing within each 20-bp window.

We decided to build motif-specific models. For each 5mer DRACH motif, we identified all occurrences of the motif within expressed transcripts. Using previously identified m^6^A sites (Linder et al. 2015; Ke et al. 2017), all occurrences of the motif were segregated into two groups of known and unknown sites. About 70% of the known occurrences were used as training data, whereas the remaining 30% of the known occurrences were used as part of the testing data. To maintain an evenness within the training data, we added the same number of unknown occurrences to the training data. Remaining unknown occurrences were added to the testing data. The known m^6^A occurrence were considered as true m^6^A sites, and the previously unidentified sites were considered as false m^6^A sites. Once the training and testing sites were identified, we extracted modification values for 10 bp upstream and downstream from the “A” within the DRACH motif. Each model was trained on these values for the given ground truth and then tested on corresponding values for the test sites.

Thus, we generated 18 RF models, each corresponding to one specific DRACH motif. Each model was trained using 10 different training data sets, and the model with the highest training accuracy was selected for testing purposes. To confirm the training accuracy, each model was tested on a test data set. To maintain the sanity of the validation, we ensured that the test data sets had not been run through the RF model in any capacity.

The purpose of the model is to identify novel m^6^A sites, in addition to the known CLIP sites. We expected the accuracy of the model to be handicapped, because many of the previously unidentified DRACH sites would now be predicted as valid m^6^A sites. Hence, the final accuracy of the model was determined as the accuracy of the model to detect previously known m^6^A sites within the test data set.

### m^6^A metagene plots

We used the metaPlotR package to plot metagene plots for m^6^A sites identified through MINES. MetaPlotR is a publicly available package (https://github.com/olarerin/metaPlotR) and has been previously used to perform similar analyses (Olarerin-George and Jaffrey 2017).

### MINES

MINES (m^6^A Identification using Nanopore Sequencing) is a command line executable code that uses a compilation of the four random models, each corresponding to a DRACH motif, AGACT, GGACA, GGACC, and GGACT. MINES uses Tombo's fraction-modified values and coverage files as inputs and outputs a bed file of predicted sites. Processing time for a full data set is ∼10 min. For more information, visit https://github.com/YeoLab/MINES.git.

## DATA DEPOSITION

MINES source code is available at https://github.com/YeoLab/MINES.git. Data files have been uploaded to GEO under accession number GSE132971.

## SUPPLEMENTAL MATERIAL

Supplemental material is available for this article.

## COMPETING INTEREST STATEMENT

G.W.Y. is cofounder, member of the Board of Directors, on the SAB, equity holder, and paid consultant for Locana and Eclipse BioInnovations. G.W.Y. is a visiting professor at the National University of Singapore and receives travel reimbursement. The terms of this arrangement have been reviewed and approved by the University of California, San Diego in accordance with its conflict of interest policies. The authors declare no other competing financial interests.

## Supplementary Material

Supplemental Material
