# Supplementary material for: Direct RNA sequencing enables m6A detection in endogenous transcript isoforms at base-specific resolution
Source: RNA. 2020 Jan;26(1):19–28. doi: 10.1261/rna.072785.119 (PMC6913132; doi:10.1261/rna.072785.119)
Supplement: Supplemental Material [file supp_26_1_19__index.html]

Direct RNA sequencing enables m6A detection in endogenous transcript isoforms at base specific resolution — Direct RNA sequencing enables m6A detection in endogenous transcript isoforms at base-specific resolution — Supplemental Material 

# Direct RNA sequencing enables m6A detection in endogenous transcript isoforms at base-specific resolution

## Supplemental Material

- Supplemental\_Materials.docx
