# Supplementary material for: Direct RNA sequencing enables m6A detection in endogenous transcript isoforms at base-specific resolution
Source: RNA. 2020 Jan;26(1):19–28. doi: 10.1261/rna.072785.119 (PMC6913132; doi:10.1261/rna.072785.119)
Supplement: Supplemental Material [file supp_072785.119_Supplemental_Materials.docx]

**SUPPLEMENTAL MATERIALS:**

**Direct RNA sequencing enables m^6^A detection in endogenous transcript isoforms at base specific resolution**

Daniel A. Lorenz, Shashank Sathe, Jaclyn M. Einstein, and Gene W. Yeo


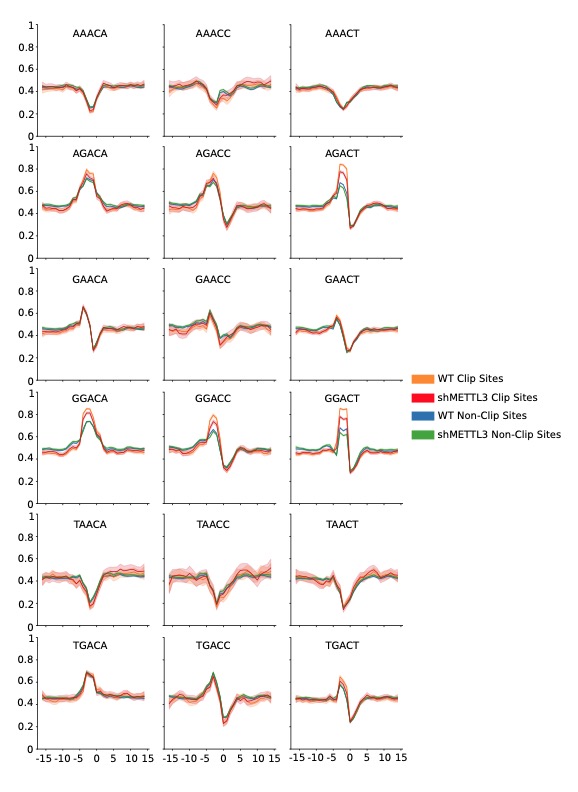


**Supplemental Figure 1.** Line plots of Tombo’s fraction modified value across a 30-nucleotide window centered on the “A” for each DRACH motif.


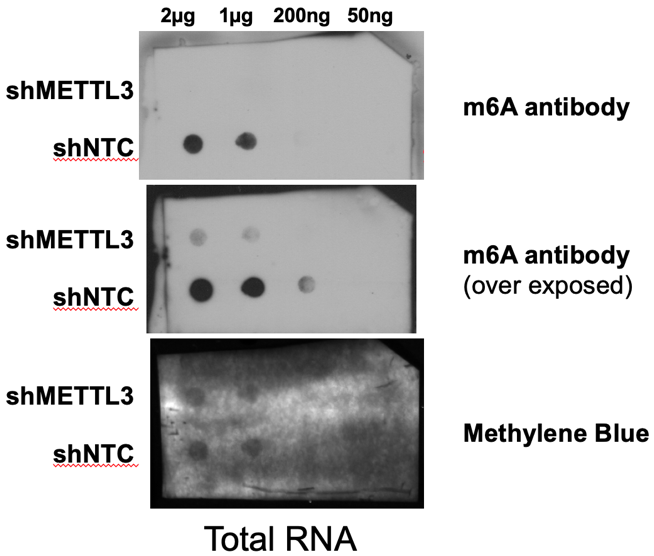


**Supplemental Figure 2.** m^6^A dot blots of total RNA isolated from HEK293T treated with non-targeting shRNA (shNTC) and shRNA targeting METTL3 (shMETTL3).

| **Motif** | **Max Accuracy** | **Mean Accuracy** | **Precision** |
| --- | --- | --- | --- |
| AAACA | 0.82 | 0.73 | 0.63 |
| AAACC | 0.76 | 0.66 | 0.63 |
| AAACT | 0.79 | 0.61 | 0.76 |
| AGACA | 0.73 | 0.64 | 0.77 |
| AGACC | 0.71 | 0.63 | 0.63 |
| AGACT | 0.72 | 0.68 | 0.89 |
| GAACA | 0.79 | 0.66 | 0.61 |
| GAACC | 0.77 | 0.62 | 0.61 |
| GAACT | 0.67 | 0.64 | 0.82 |
| GGACA | 0.82 | 0.69 | 0.92 |
| GGACC | 0.75 | 0.66 | 0.86 |
| GGACT | 0.83 | 0.78 | 0.91 |
| TAACA | 0.73 | 0.61 | 0.63 |
| TAACC | 0.68 | 0.52 | 0.4 |
| TAACT | 0.72 | 0.56 | 0.59 |
| TGACA | 0.67 | 0.59 | 0.57 |
| TGACC | 0.71 | 0.58 | 0.57 |
| TGACT | 0.74 | 0.65 | 0.81 |

**Supplemental Table 1.** Table of accuracy and precision values. Highlighted are the four motifs which met our >70% maximum accuracy and >85% precision.

**Supplemental Figure 3.** ROC curves for each DRACH motif.


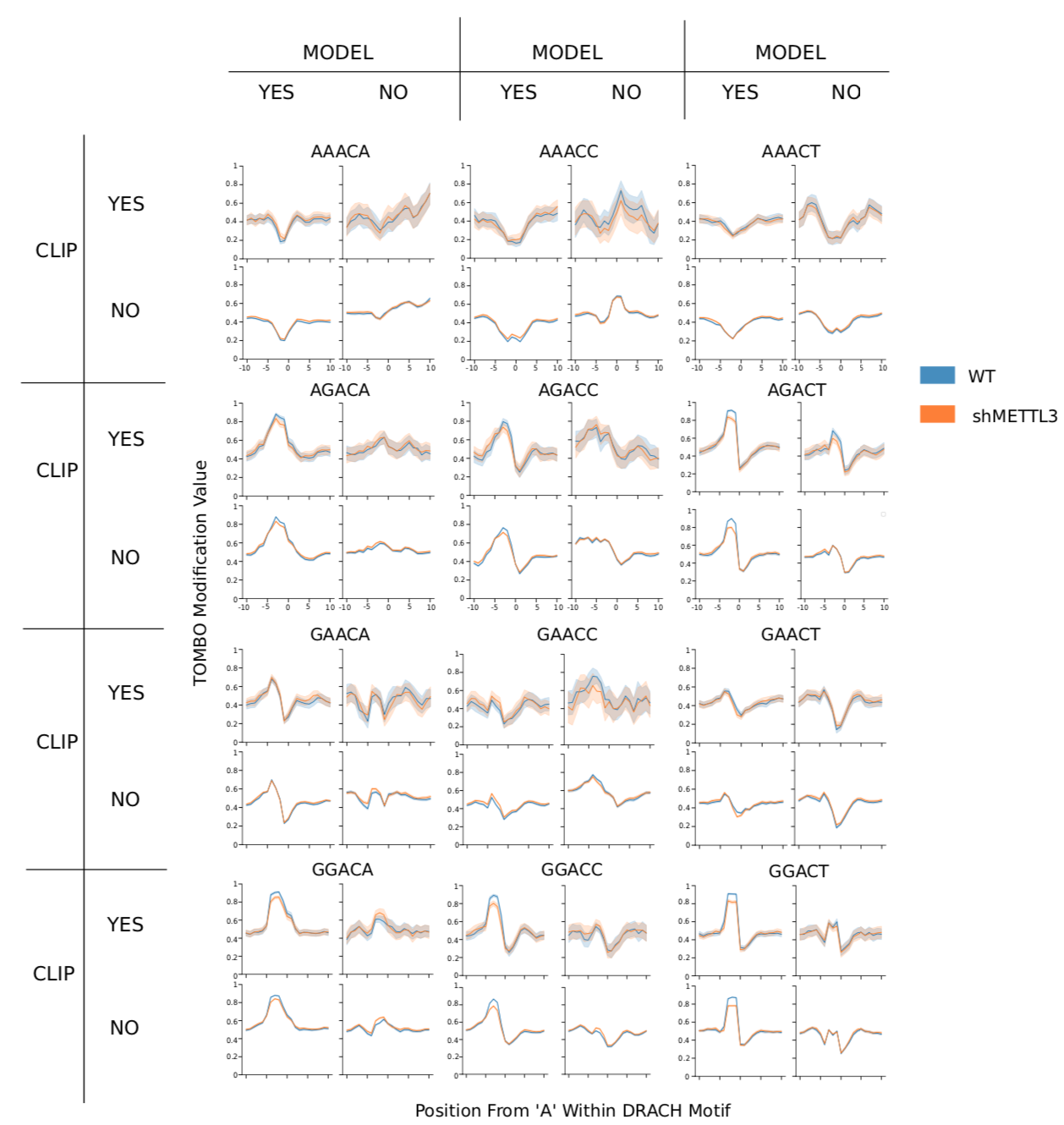


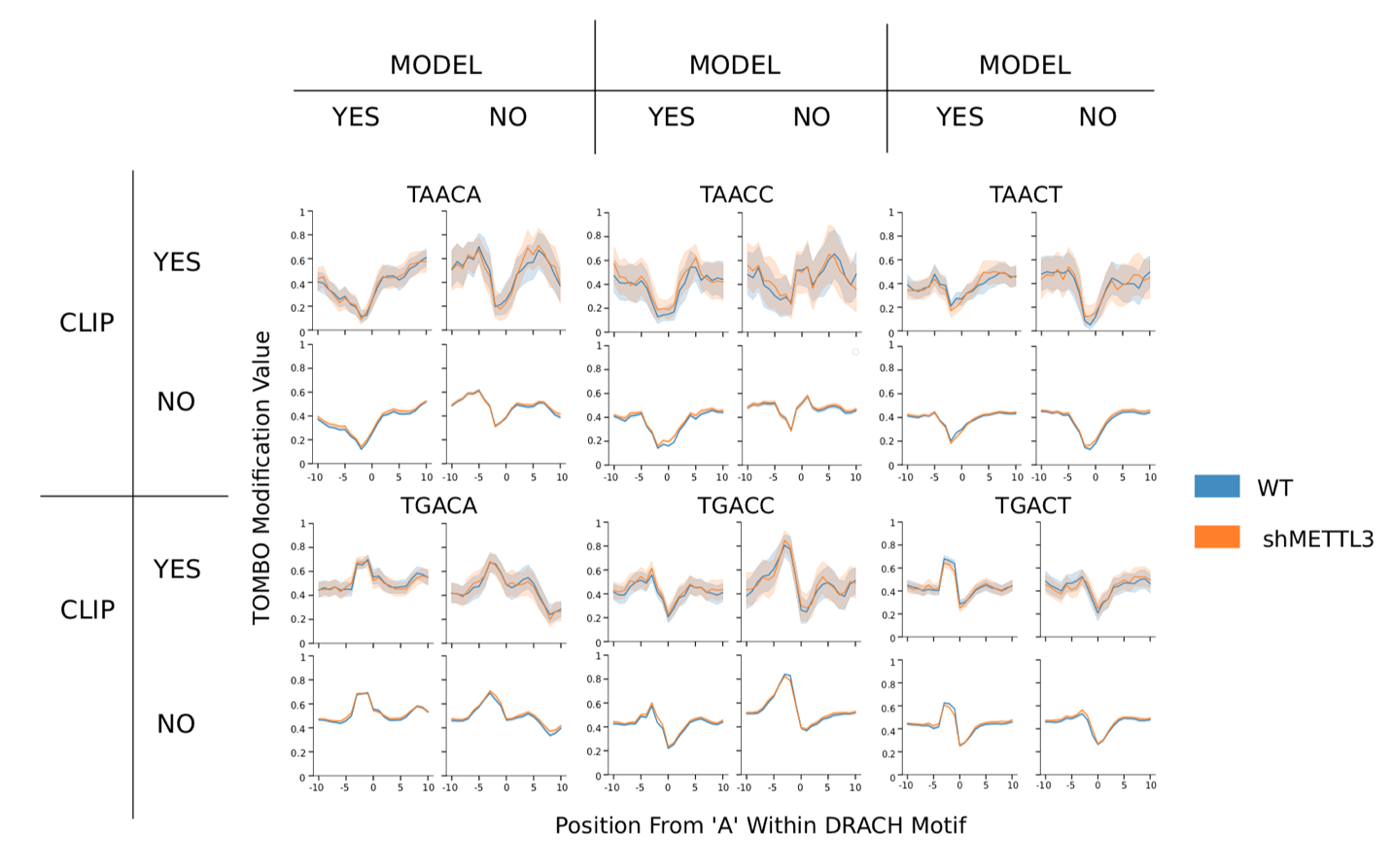


**Supplemental Figure 4.** Line plots of Tombo’s fraction modified values broken down by CLIP sites and model predictions in HEK293T for each DRACH motif.


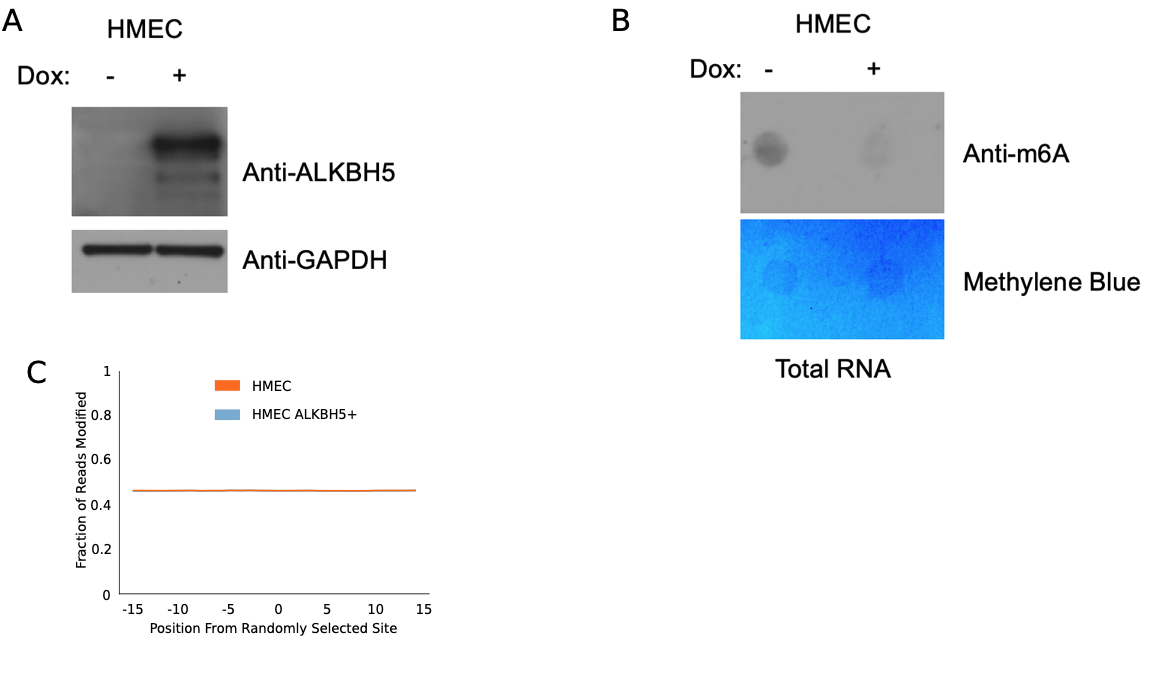


**Supplemental Figure 5.** (**A**) Western blots depicting ALKBH5 overexpression in HMEC. (**B**) m^6^A dot blots of total RNA isolated from wild-type HMEC (-) and HMEC overexpressing ALKBH5 (+). (**C**) Line plot of Tombo’s fraction modified values in HMEC for randomly shuffled sites.


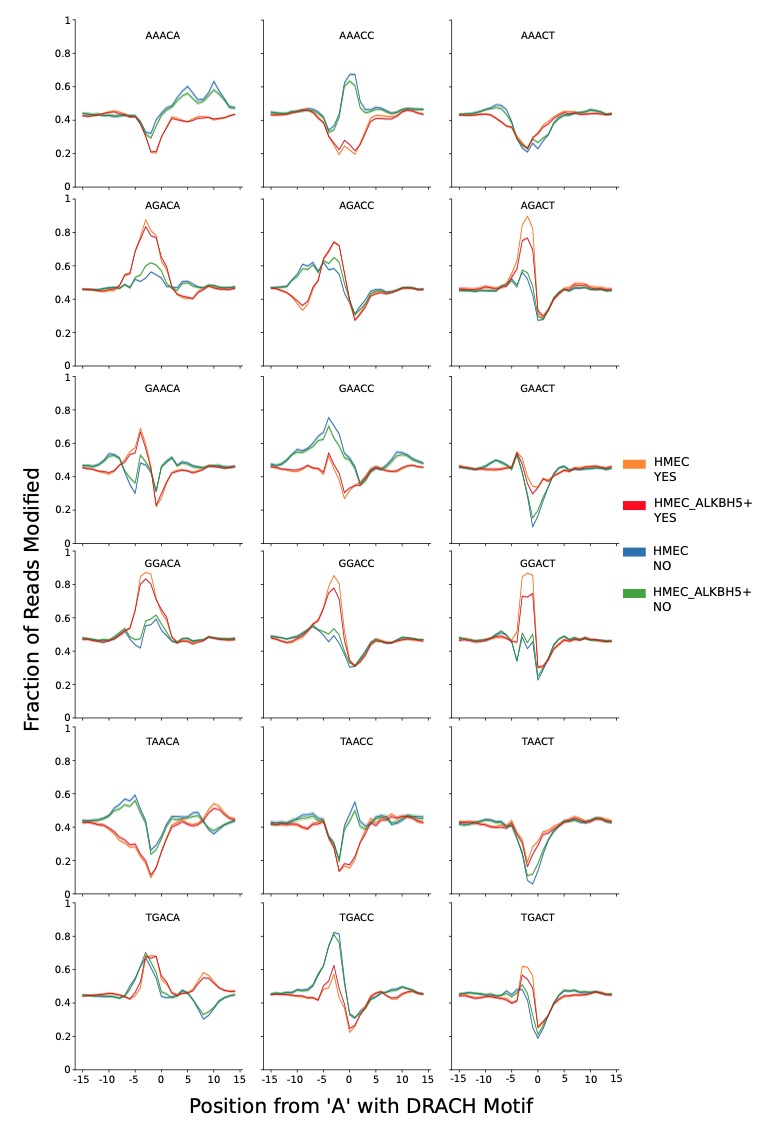


**Supplemental Figure 6.** Line plot of Tombo’s fraction modified values in HMEC for each DRACH motif.
